# *Leucaena leucocephala* Fruit Aqueous Extract Stimulates Adipogenesis, Lipolysis, and Glucose Uptake in Primary Rat Adipocytes

**DOI:** 10.1155/2014/737263

**Published:** 2014-08-10

**Authors:** Umah Rani Kuppusamy, Bavani Arumugam, Nooriza Azaman, Chai Jen Wai

**Affiliations:** ^1^Department of Biomedical Science, Faculty of Medicine, University of Malaya, 50603 Kuala Lumpur, Malaysia; ^2^Faculty of Medicine, Segi University, Selangor 47810, Malaysia

## Abstract

*Leucaena leucocephala* had been traditionally used to treat diabetes. The present study was designed to evaluate *in vitro* “insulin-like” activities of *Leucaena leucocephala* (Lam.) deWit. aqueous fruit extract on lipid and glucose metabolisms. The ability of the extract to stimulate adipogenesis, inhibit lipolysis, and activate radio-labeled glucose uptake was assessed using primary rat adipocytes. Quantitative Real-Time RT-PCR was performed to investigate effects of the extract on expression levels of genes (protein kinases B, AKT; glucose transporter 4, GLUT4; hormone sensitive lipase, HSL; phosphatidylinositol-3-kinases, PI3KA; sterol regulatory element binding factor 1, Srebp1) involved in insulin-induced signaling pathways. *L. leucocephala* aqueous fruit extract stimulated moderate adipogenesis and glucose uptake into adipocytes when compared to insulin. Generally, the extract exerted a considerable level of lipolytic effect at lower concentration but decreased gradually at higher concentration. The findings concurred with RT-PCR analysis. The expressions of GLUT4 and HSL genes were upregulated by twofold and onefold, respectively, whereas AKT, PI3KA, and Srebp1 genes were downregulated. The *L. leucocephala* aqueous fruit extract may be potentially used as an adjuvant in the treatment of Type 2 diabetes mellitus and weight management due to its enhanced glucose uptake and balanced adipogenesis and lipolysis properties.

## 1. Introduction

Type 2 diabetes mellitus (Type 2 DM) is recognized as the most prevalent type of diabetes. Occurrence of Type 2 DM is primarily related to the inability of glucose uptake into tissues due to defects in insulin action, a phenomenon known as insulin resistance. Insulin stimulates rapid translocation of a major insulin-sensitive transporter, GLUT4, which transports glucose from the intracellular storage milieu to the plasma membrane. Resistance to this effect causes an increase in blood glucose level, a condition known as hyperglycemia [1, 2]. Critically, individuals with hyperglycemia could progressively develop other major life-threatening or debilitating complications such as cardiomyopathy (CVD), retinopathy, nephropathy, neuropathy, amputations, obesity, and hypertension [3, 4].

Numerous plant extracts and their derivatives were discovered to offer potential treatment for diabetes [5–7] with minimal side effects compared to modern antidiabetic or glucose-lowering drugs [8, 9].* Leucaena leucocephala* (Lam.) deWit. classified in the family Leguminosae is commonly known as “*petai belalang*” by Indonesians and Malaysians. In these countries, the fruit is eaten raw as “*ulam*” the same way as* Parkia speciosa Hassk* (“Petai”), classified under the same family.* L. leucocephala* tree has a wide range of applications and was once referred to as the “miracle tree” due to its medicinal efficacy. The* L. leucocephala* bark extract is consumed to treat internal pain while the root and bark are taken as a contraceptive or depilatory in Latin America [10].* L. leucocephala* had been traditionally used to treat diabetes, especially by Indonesians and Mexicans [11]. Indonesians consume the aqueous extract derived from boiled seeds orally to treat diabetes.* Leucaena* seeds showed hypoglycemic effect on alloxan-induced diabetic mice [12]. In a separate study, seed extracts of* L. leucocephala* lowered blood glucose and lipid levels and increased regeneration of *β*-cells of the pancreas in streptozotocin-induced diabetic rats [13]. Recently, ethanolic extract of seeds of* L. leucocephala* was reported to actively inhibit *α*-glucosidase activity [14]. Meanwhile, glycoside compounds with monosaccharide galactose clusters identified in the methanolic seed extract of the plant showed antihyperglycemic activities in alloxan-induced diabetic rats [15].

To date, the effect of aqueous extract from fruit of* L. leucocephala* on glucose homeostasis has not been reported. Therefore, this study was designed to evaluate “insulin-like” effect of* L. leucocephala* aqueous fruit extract on the lipid and glucose metabolisms by assessing the ability of the extract to stimulate adipogenesis and glucose uptake as well as inhibiting lipolysis in primary rat adipocytes. In addition, gene expression study was performed to further validate the extract induced-alteration in the selected insulin pathway regulatory genes.

## 2. Materials and Methods

### 2.1. Reagents

Penicillin-streptomycin-glutamine solution and insulin were purchased from Invitrogen (California, USA). Isobutylmethylxanthine (IBMX), glycerol kinase, glycerol phosphate oxidase, dexamethasone, adenosine-5-triphosphate (ATP), and liquid scintillation cocktail (Cytoscint) were supplied by ICN (Ohio, USA). Type-II collagenase and tritiated deoxyglucose (2-deoxy-D-[2,6-^3^H] glucose) were procured from Worthington (New Jersey, USA) and Perkin Elmer (Massachusetts, USA), respectively. Phosphate buffered saline (PBS) tablets and amphotericin B (Fungizone) were obtained from Flow Lab, Australia. Tissue culture media, supplements, chemicals, and reagents were purchased from Sigma Chemical Co., USA, unless otherwise stated. TaqMan gene expression assay kits were obtained from Applied Biosystems (California, USA). All other reagents used were of analytical grade. All the experiments were performed at 28°C unless otherwise stated.

### 2.2. Preparation of* Leucaena leucocephala* Extract

*L. leucocephala* fruits were washed with distilled water, ovendried at 40°C, and powdered. Sterile distilled water (1000 mL) was added to 50 g of the powdered fruits in a flask and stirred for 3 hours at 40°C. This mixture was centrifuged at 3000 ×g for 10 minutes and the resulting supernatant was freeze-dried (aqueous extraction of the powderized* L. leucocephala* fruit resulted in 13% yield of extract) and stored at −20°C for subsequent use. The residue was reconstituted with sterile ultrapure water. The reconstituted extract was filter sterilized with syringe filters (0.2 *μ*m) prior to incubation with adipocytes and subsequent adipogenesis, lipolysis, and glucose uptake assays.

### 2.3. Cell Isolation, Culture, and Differentiation

Male* Sprague-Dawley* rats of 6–8 weeks old were sacrificed by cervical dislocation. The intra-abdominal fat pads were excised and the preadipocytes were isolated according to the method described by Bjorntorp and coworkers [16]. Collagenase digestion was done at 37°C for an hour and the harvested cells were cultured in RPMI-1640 supplemented with 2 mM penicillin-streptomycin-glutamine, 2 mM amphotericin B, and 10% foetal bovine serum (FBS) at 37°C and 5.0% CO_2_. The medium was routinely changed every 2-3 days until the cells reached a confluent state. In some parts of the study, preadipocytes were cultured in 12-well plates. Induction of differentiation was done on Day 0 using DMEM supplemented with 2 mM penicillin-streptomycin-glutamine, 2 mM amphotericin B, 10% FBS, 33 *μ*M biotin, 17 *μ*M pantothenic acid, 10 *μ*M troglitazone, 0.5 mM IBMX, 1.0 *μ*M dexamethasone, and 10 *μ*g/mL insulin (part-1 differentiation media, DM 1) at 37°C and 5.0% CO_2_. Dexamethasone and IBMX were used to induce the differentiation process besides insulin as the reagents stimulate glucocorticoid receptor and cAMP-dependent protein kinase pathways, respectively [17]. Troglitazone, a member of thiazolidinediones (TZD), was used as the insulin sensitizing agent to induce preadipocyte differentiation via activation of PPAR gamma [18]. From Day 2 onwards, the cells were cultured in similar medium but free of troglitazone, IBMX, and dexamethasone (part-2 differentiation media, DM 2) until full differentiation was achieved [19]. Experiments were carried out on Day 9–Day 12, at optimum differentiation state when cells are considered as mature adipocytes (optimum percentage of lipid accumulation varies and depends on the need in the subsequent assays).

### 2.4. Adipogenesis Assay

Various concentrations of* L. leucocephala* extract were added together with DM 2 as described earlier in the differentiation protocol in the presence or absence of insulin (insulin 100 *μ*M as a positive control). On Day 10 of differentiation, cells were washed twice with PBS, fixed in 0.5% formalin, and rinsed with 60% isopropanol before staining with Oil Red O solution (60%) [20, 21]. Cells were washed twice with PBS in order to remove excess dye. Spectrophotometric quantification was done by dissolving the Oil Red O absorbed by lipid droplets with 100% isopropanol at 510 nm using a microplate reader. The extent of adipogenesis was expressed as follows:

(1) $$\begin{array}{c} \text{Adipogenic}\;\text{activity}\;\left(\text{fold}\;\text{increase}\right)=\frac{A_{\text{sample}}-A_{\text{control}}\;}{\;A_{\text{control}}}. \end{array}$$

“Control” and “sample” refer to untreated cells and cells treated with extracts or insulin, respectively.

### 2.5. Lipolysis Assay

Lipolysis in adipocytes was quantified using glycerol quantification assay as described earlier [21, 22]. On Day 12 of differentiation, cells were cultured in serum-free medium for 24 hours and incubated for 5 hours with (a) epinephrine (1 *μ*M) as a positive lipolysis control, (b) insulin (l *μ*M) as antilipolysis control, (c) insulin and epinephrine, (d) various concentrations of the extract, (e) extract of different concentrations and epinephrine, (f) extract of different concentrations and insulin, and (g) extract of different concentrations, insulin and epinephrine. Glycerol released from triglycerides during lipolysis was spectrophotometrically quantified at 540 nm using a microplate reader upon incubation of the cultures with glycerol quantification reagent for 15 minutes at 37°C. Increase in amount of glycerol formed in the sample is directly proportional to the increase in lipolysis. The extent of lipolysis was expressed in fold increase over untreated cells.

### 2.6. Glucose Uptake Assay

Glucose uptake activity of the cells was determined according to the method recommended by Chai and coworkers using radioactive-labeled glucose [21]. Cells were treated with* L. leucocephala* aqueous fruit extracts of various concentrations, with insulin (100 *μ*M) and ultrapure water as positive and basal control, respectively. The amount of 2-deoxy-D-[2,6-^3^H] glucose taken up by adipocytes was quantified through scintillation counting and was expressed as counts per minute (c.p.m.). The relative glucose uptake was expressed as the percentage differences between insulin-stimulated glucose uptake and the basal glucose uptake divided by basal glucose uptake.

### 2.7. Gene Expression Assessment

On Day 12 after the initiation of differentiation, the spent media were aspirated and replaced with media containing treatment compounds. Insulin (final concentration of 10 *μ*g/mL) was used as a positive control; meanwhile ultrapure water was used as a negative control. The cells were incubated for 24 hours before total RNA was extracted using a commercialized spin cartridge kit (Invitrogen). Purity and quantity of RNA were estimated. The purified RNA was immediately subjected to reverse transcription to synthesize first-strand cDNA by using a high capacity cDNA reverse transcription kit (Applied Biosystems) according to manufacturer's protocol.

Quantitative Real-Time reverse transcription-polymerase chain reaction (RT-PCR) was performed to analyze gene expression levels using reaction setup manual provided by the StepOnePlus Real-Time PCR system (Ver 2.0, Applied Biosystems) and TaqMan probes.  Table 1 shows a list of genes and their respective accession numbers investigated in this study. Each reaction was performed in triplicate. Relative gene expression was determined by the comparative Ct method, also known as 2^−ΔΔCt^ method [23]. Expression of the genes was normalized to eukaryotic 18S rRNA (endogenous control). Results were expressed as* n*-fold difference over respective controls. Fold variation values less than 0 were expressed as negative values. For statistical analyses of Real-Time RT-PCR experiments, gene expression results were expressed as a difference in the ΔCt value obtained between treated versus untreated cells.

### 2.8. Statistical Analyses

All experiments were carried out in quadruplicate and values were expressed as mean ± SEM, unless otherwise stated. The statistical significance was evaluated by Student's *t*-test and ANOVA. In statistical analysis using the GraphPad Prism version 5.02, significance comparisons, ∗ denotes *P* < 0.05, ∗∗ denotes *P* < 0.01, and ∗∗∗ denotes *P* < 0.001.

## 3. Results

### 3.1. Effect of* Leucaena leucocephala* Fruit Extract on Adipogenesis

**Figure 1:**
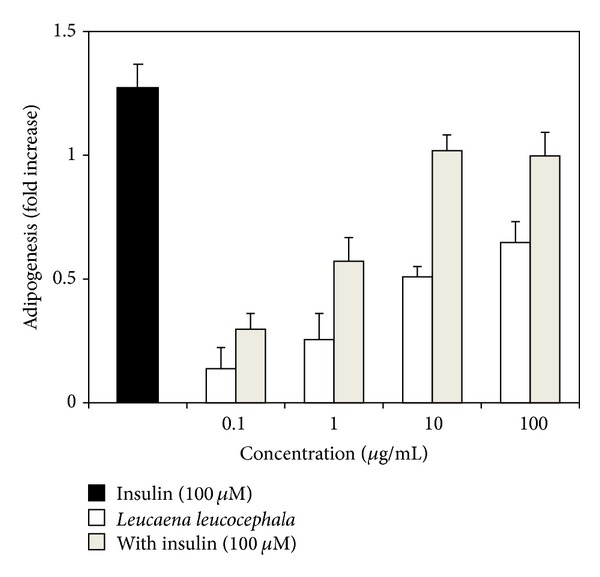
Effect of* Leucaena leucocephala* aqueous fruit extract.* y*-axis indicates the fold increase of adipogenesis ± SEM of quadruplicate assays over control value (untreated cells). Preadipocytes (80,000 cells/well) were induced to differentiate with various concentrations of* Leucaena leucocephala* fruit extracts with/without insulin (100 *μ*M) prior to Oil Red O assay.

Figure 1 shows the fold increase of lipid content in mature adipocytes, as determined by Oil Red O quantification assay after the treatment of the cells with* L. leucocephala* extract in the absence or presence of insulin. Insulin (100 *μ*M), served as the positive control, enhanced the adipogenic activity in rat adipocytes, efficiently*. L. leucocephala* fruit extract (in the absence of insulin) showed a mild adipogenic activity which improved with increasing concentration of the extract (0.1–100 *μ*g/mL).

### 3.2. Effect of* Leucaena leucocephala* Fruit Extract on Lipolysis

**Figure 2:**
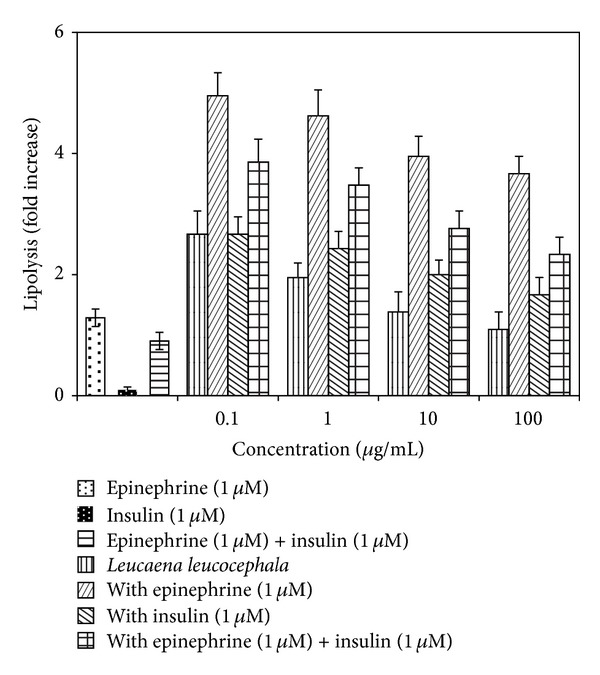
Effect of* Leucaena leucocephala* aqueous fruit extract on lipolysis.* y*-axis indicates the fold increase of epinephrine induced lipolysis ± SD of quadruplicate assays over control value (untreated cells). Adipocytes (80,000 cells/well) were treated with various concentrations of* Leucaena leucocephala* fruit extracts, epinephrine (1 *μ*M), and/or insulin (1 *μ*M) prior to glycerol quantification assay.

Epinephrine, an *α*- and *β*-adrenergic agonist, activates hormone sensitive lipase (HSL) to stimulate lipolysis by promoting adenylate cyclase activity [24].* L. leucocephala* extract at a concentration range of 0.1−100 *μ*g/mL showed a dose-dependent decreasing lipolytic activity in fully differentiated adipocytes (Figure 2). The extract exerted higher or equivalent lipolytic effect compared to epinephrine (1 *μ*M) induced lipolysis throughout the range of concentration. This effect was further enhanced upon coincubation of the extract with epinephrine (1 *μ*M) but decreased with increasing concentration (0.1–100 *μ*g/mL) of the extract.

Insulin is well known to have antilipolytic property [25] and therefore showed considerable attenuation of epinephrine (1 *μ*M) induced lipolysis. Nevertheless, insulin could not attenuate extract (0.1–100 *μ*g/mL) or epinephrine (1 *μ*M) and extract (0.1–100 *μ*g/mL) induced lipolysis. However, the lipolytic effect of* L. leucocephala* fruit extract decreased and was comparable to the effect of insulin on epinephrine-induced lipolysis at an extract concentration of 100 *μ*g/mL.

### 3.3. Effect of* Leucaena leucocephala* Fruit Extract on Glucose Uptake

**Figure 3:**
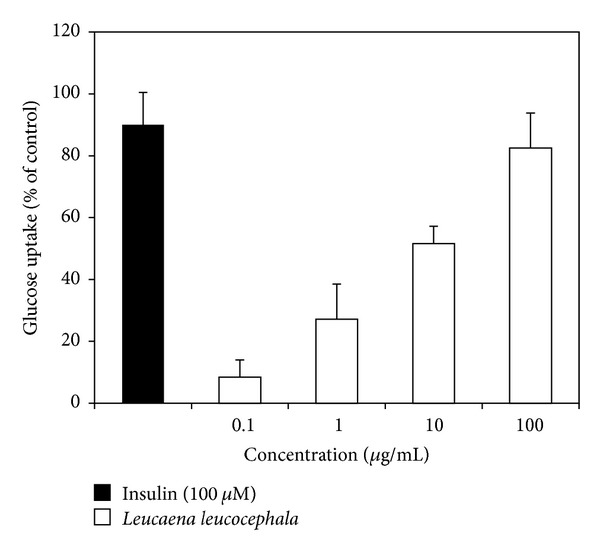
Effect of* Leucaena leucocephala* aqueous fruit extract and insulin on glucose uptake in rat adipocytes.* y*-axis indicates the mean percentages of glucose uptake ± SEM of quadruplicate assays over control value (untreated cells). Adipocytes (80,000 cells/well) were treated with various concentrations of* Leucaena leucocephala* extracts or insulin (100 *μ*M) prior to radio-labeled glucose uptake assay.

In the radio-labeled glucose uptake study,* L. leucocephala* fruit extract showed an enhanced glucose uptake activity upon increasing concentration (0.1−100 *μ*g/mL) of the extract (Figure 3). The glucose uptake effect of the extract at a concentration of 100 *μ*g/mL was comparable to insulin (100 *μ*M).

### 3.4. Effect of* Leucaena leucocephala* Fruit Extract on Gene Expression

**Figure 4:**
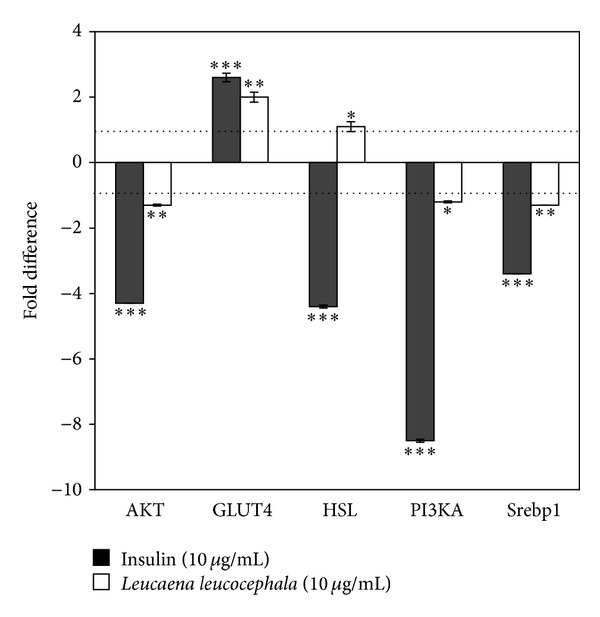
Effect of* Leucaena leucocephala* aqueous fruit extract on AKT, GLUT4, HSL, PI3KA, and Srebp1 gene expressions.* y*-axis represent the fold difference (mean ± SD) of* Leucaena leucocephala* (10 *μ*g/mL) stimulated gene expression over control compared to insulin (10 *μ*g/mL). Eukaryotic 18S rRNA was used as the endogenous control. Dotted lines represent variation between +1 and −1 which corresponds to no change. Significance comparisons: ∗ denotes *P* < 0.05, ∗∗ denotes *P* < 0.01, and ∗∗∗ denotes *P* < 0.001 versus control.

Figure 4 shows fold difference of* L. leucocephala* fruit extract or insulin-induced gene expressions.* L. leucocephala* fruit extract at 10 *μ*g/mL corresponded to 50% effective concentration (EC_50_) particularly in stimulating adipogenesis and glucose uptake activities. Hence, the concentration, 10 *μ*g/mL of the extract, was utilized for the gene expression assay. Insulin (10 *μ*g/mL) as the positive control upregulated GLUT4 gene and downregulated AKT, HSL, PI3KA, and Srebp1 genes compared to the untreated cells in the present study. Similarly, the extract also stimulated GLUT4 gene expression (*P* < 0.01) and downregulated expression of AKT (*P* < 0.01), PI3K (*P* < 0.05), and Srebp1 (*P* < 0.01) genes. Insulin, as an antilipolytic agent, downregulated HSL gene expression by 4.4-fold, whereas,* L. leucocephala* fruit extract upregulated expression of HSL by onefold (*P* < 0.05) (Figure 4).

## 4. Discussion

Adipocytes model is emerging as a potential therapeutic target in diabetes as well as in obesity research [26]. Insulin is capable of inducing adipogenesis, suppressing lipolysis, and stimulating the uptake of glucose and free fatty acids in adipose besides liver and muscle tissues [25]. Insulin lowers blood glucose level by transporting glucose from intracellular stores to the plasma membrane of adipocytes upon activation of insulin-signalling pathway [27]. Hence, plant extracts with insulin-like and/or insulin-sensitizing properties could serve as adjuvants for the treatment of DM.

Primary rat preadipocytes were differentiated to mature adipocytes for the assessment of adipogenesis, antilipolysis, and glucose uptake stimulatory effects of* L. leucocephala* aqueous fruit extract. The findings obtained from the adipogenesis assay suggest that* L. leucocephala* fruit extract was able to activate adipogenesis, which reflects an “insulin-like” characteristic (Figure 1).

The extract exhibited lipolytic activity at lower concentrations. The lipolytic effect of* L. leucocephala* extract was not affected by insulin (Figure 2), which is in line with findings observed with genistein and *β*-sitosterol (SIT) [21, 28] and is speculated to be the result of activation of protein kinase A. Nevertheless, lipolytic effect of* L. leucocephala* fruit extract progressively decreased with increasing concentration of the extract (Figure 2). Although antilipolysis resembles “insulin-like” property, it could raise problems with weight gain as it inhibits lipid mobilization from adipocytes. Insulin and sulfonylureas therapies help lower plasma blood glucose level but accelerate weight gain in Type 2 DM patients [29]. Thus, “insulin-like” plant extracts which show mild lipolysis or antiadrenaline induced lipolytic activity could serve as a better agent to treat diabetes. For instance,* Lagerstroemia speciosa* L. extracts which enhanced glucose uptake in 3T3-L1 adipocytes but attenuated adipogenesis were proposed as a potential hypoglycemic agent [30]. Interestingly, the* L. leucocephala* fruit extract enhanced glucose uptake into the rat adipocyte efficiently (Figure 3), which is in line with its adipogenesis effect (Figure 1). Nevertheless, the extract also exhibited lipolytic activity at all the concentrations tested though its lipolytic activity reduced with increasing concentration of the extract. Hence, it can be hypothesized that the extract with enhanced glucose uptake as well as balanced lipolysis and adipogenesis activities, for instance, at an ideal concentration (such as 100 *μ*g/mL in the present study), could serve as a better “insulin-like” agent with less weight gain effects similar to metformin. The latter is a recommended therapy for the management of hyperglycemia in overweight diabetic patients as it also promotes weight loss [29]. In addition, plant extracts with high lipolytic effect are desirable for the development of antiobesity drugs. Several plants such as citrus fruit [31],* Curcuma longa* [32], and* Nelumbo nucifera* leaves [33] have been shown to exhibit lipolytic effect.* L. leucocephala* also has a great potential to be used in antiobesity treatment due to its strong lipolytic activity at lower concentrations.

In order to further validate the “insulin-like” potential of* L. leucocephala* fruit extract, expression levels of several genes, that is, protein kinases B (AKT), glucose transporter 4 (GLUT4), hormone sensitive lipase (HSL), phosphatidylinositol-3-kinases (PI3KA), and sterol regulatory element binding factor 1 (Srebp1) (Table 1), were investigated. Insulin lowers blood glucose level by increasing the transport of glucose into adipose and muscle tissues [34]. This involves insulin-induced rapid translocation of glucose transporter, GLUT4, from intracellular to the plasma membrane through the insulin signaling cascade. Insulin binds to the insulin receptor and activates insulin receptor substrates (IRS 1) through autophosphorylation. The reaction is mediated by tyrosine kinase. This is followed by sequential phosphorylation of PI3KA, phosphoinositide-dependent kinase-1 (PDK-1), and downstream effector AKT/PKB (protein kinase B). This eventually leads to translocation of GLUT4 to ease uptake of glucose into cells [35]. Insulin also plays a significant role in preadipocyte differentiation (adipogenesis) by triggering expression of transcription factors, that is, PPAR gamma, which results in activation of insulin signaling cascade and hence induction of GLUT4 [36]. Adipogenesis and lipolysis are regulated by insulin and epinephrine pathways, respectively. Epinephrine triggers *β*-adrenergic receptors mediated activation of adenylyl cyclase to produce cAMP. This leads to activation of protein kinase A (PKA) followed by HSL to initiate lipid mobilization (lipolysis). On the other hand, insulin inhibits the effect of epinephrine by breaking cAMP's phosphodiesterase bond through phosphorylation of phosphodiesterase [37].

Upregulation of GLUT4 gene clearly reflects the ability of both insulin and* L. leucocephala* extract to enhance glucose uptake into adipocytes by 2.5-fold and 2.0-fold, respectively (Figures 3 and 4). PPAR gamma-induced adipogenesis followed by the stimulation of the insulin signaling cascade [36] correlates well with the stimulation of adipogenesis activity (Figure 1) and enhancement of glucose uptake/GLUT4 gene expression (Figures 3 and 4) displayed by both insulin and* L. leucocephala* extract. Conversely, downregulation of AKT, PI3KA, and Srebp1 gene expression (Figure 4) could possibly be regulated at the posttranslational level to manage glucose uptake into the cells [21].

Besides, upregulation of HSL gene by the extract correlated well with its lipolytic property (Figure 2). The extract could have triggered the cAMP pathway to activate PKA and HSL, thus leading to lipid mobilization as similar to the mechanism exerted by epinephrine. Nevertheless, the extract inhibited lipolysis in the cell at higher concentrations, possibly due to improved adipogenesis property as well as activation of insulin pathway. PI3KA also plays a vital role in preadipocyte differentiation to adipocytes. Lipid accumulation in 3T3-L1 cells was reported to decrease when the cells were treated with PI3KA inhibitor [38]. Meanwhile, Srebp1, transcription factors which are highly found in adipose tissue, also promotes adipogenesis by controlling endogenous ligand to activate PPAR gamma [39]. In the present study, downregulation of AKT, PI3KA, and Srebp1 genes may predominantly be associated with the greater lipolytic effect exerted by* L. leucocephala* aqueous fruit extract than its adipogenic activity in the rat adipocytes at a dose of 10 *μ*g/mL, which was used for gene expression assay in the present study (Figures 2 and 4).

Numerous plants and their derivatives with high content of natural antioxidants offer a prudent option for the treatment of diabetes [5–7]. For instance,* Catharanthus roseus* (*Sadabahar*) [40],* Trigonella foenum-graceum* L (fenugreek) [41],* Momordica charantia* L. Var.* abbreviata* Ser (bitter melon) [42], and* Syzygium aqueum* (water apple) [43] were reported to contain various flavonoids, insulin-like polypeptide, and alkaloids that have antidiabetic properties. The* L. leucocephala* plant extract was shown to contain carotenoids, phenolic compounds, vitamin E, and high vitamin C content in a previous report [44]. Recently, Chew and coworkers reported the presence of tannin, flavonoid, steroid, saponin, and alkaloid particularly mimosine in leaf and fruit extracts of the plant [45]. It could be speculated that the naturally occurring bioactive compounds in* L. leucocephala* extracts could contribute to its “insulin-like” property as revealed by various glucose and lipid metabolism assessments performed in the present study.

## 5. Conclusion

*L. leucocephala* aqueous fruit extract was able to activate adipogenesis and glucose uptake in rat primary adipocytes efficiently, which correlated well with findings obtained in the gene expression study. The extract showed an inverse relationship between adipogenesis and lipolysis. It exerted lipolytic effect on fully differentiated adipocytes but this effect decreased gradually as the concentration of the extract was increased. It can be proposed that the extract at an ideal concentration with increased glucose uptake as well as balanced adipogenesis and lipolytic effects would be valuable in the management of Type 2 DM. Further investigations are needed to identify bioactive compounds present in the aqueous fruit extract and their mechanism of action in exerting “insulin-like” and/or “antiobese” potentials.

## Figures and Tables

**Table 1:** List of probes used in Real-Time PCR.

Gene name	Gene symbol/gene aliases	Assay ID	Accession number
Protein kinase B	AKT1/AKT	Rn00583646_m1	NM_033230.1
Sterol regulatory element binding factor 1	Srebf1/Srebp1	Rn01495772_g1	AF286470.2
Hormone sensitive lipase	Lipe/HSL	Rn00689222_m1	NM_012859.1
Phosphatidylinositol-3-kinase	Pik3r1/PI3KA	Rn00564547_m1	NM_013005.1
Solute carrier family 2 (facilitated glucose transporter), member 4	Slc2a4/GLUT4	Rn00562597_m1	NM_012751.1

General abbreviation of the genes and their respective assay ID and accession numbers were obtained from the Applied Biosystems website. The assay ID indicates “Applied Biosystems gene expression assays” inventoried kits with patented primer and TaqMan probe mix. Assay ID with “Rn” prefix is defined as “*Rattus norvegicus.*” All gene expression assay kits listed here are FAM/MGB probed. Eukaryotic 18S rRNA was used as an endogenous control.
